# Insulin Like Growth Factor-1 (IGF-1) Causes Overproduction of IL-8, an Angiogenic Cytokine and Stimulates Neovascularization in Isoproterenol-Induced Myocardial Infarction in Rats

**DOI:** 10.3390/ijms12128562

**Published:** 2011-11-29

**Authors:** Nagaraja Haleagrahara, Srikumar Chakravarthi, Lisa Mathews

**Affiliations:** 1 Faculty of Medicine, Health and Molecular Sciences, James Cook University, Townsville 4811, Queensland, Australia; 2 Division of Pathology, International Medical University, Kuala Lumpur 57000, Malaysia; E-Mail: srikumar_chakravarthi@imu.edu.my; 3 Division of Human Biology, International Medical University, Kuala Lumpur 57000, Malaysia; E-Mail: lisam812@hotmail.com

**Keywords:** IGF-1, isoproterenol, myocardial ischemia, angiogenesis, interleukin-8

## Abstract

Angiogenesis factors are produced in response to hypoxic or ischemic insult at the site of pathology, which will cause neovascularization. Insulin like growth factor-1 (IGF-1) exerts potent proliferative, angiogenic and anti-apoptotic effects in target tissues. The present study was aimed to evaluate the effects of IGF-1 on circulating level of angiogenic cytokine interleukin-8 (IL-8), in experimentally-induced myocardial ischemia in rats. Male Sprague-Dawley rats were divided into control, IGF-1 treated (2 μg/kg/day subcutaneously, for 5 and 10 days), isoproterenol (ISO) treated (85 mg/kg, subcutaneously for two days) and ISO with IGF-1 treated (for 5 and 10 days). Heart weight, serum IGF-1, IL-8 and cardiac marker enzymes (CK-MB and LDH) were recorded after 5 and 10 days of treatment. Histopathological analyses of the myocardium were also done. There was a significant increase in serum cardiac markers with ISO treatment indicating myocardial infarction in rats. IGF-1 level increased significantly in ISO treated groups and the level of IGF-1 was significantly higher after 10 days of treatment. IL-8 level increased significantly after ISO treatment after 5 and 10 days and IGF-1 concurrent treatment to ISO rats had significantly increased IL-8 levels. Histopathologically, myocyte necrosis and nuclear pyknosis were reduced significantly in IGF-1 treated group and there were numerous areas of capillary sprouting suggestive of neovascularization in the myocardium. Thus, IGF-1 protects the ischemic myocardium with increased production of circulating angiogenic cytokine, IL-8 and increased angiogenesis.

## 1. Introduction

Angiogenesis is a process involving the formation of new blood vessels from pre-existing vasculature. It occurs in many physiological and pathological conditions [1]. Although neovascularization is an important vascular response to chronic hypoxia, the role of angiogenesis in myocardial ischemia is not clear yet [2,3]. Therapeutic angiogenesis is being tested as a novel treatment for ischemic heart disease [4]. In the last decennium, the challenge to research has been to find methods of inducing new vascular growth in the ischemic myocardium due to atherosclerotic coronary artery disease, which could not be treated with balloon angioplasty or coronary by-pass grafting. Therapeutic angiogenesis with recombinant vascular endothelial growth factor proteins or gene encoding for the proteins is a new potential treatment for cardiovascular disease [5]. Insulin like growth factor-1 (IGF-1) is a peptide hormone structurally related to insulin which has a pleiotropic effect on cell growth and metabolism. The biological actions attributed to IGF-1 include chemo-attractant properties, ability to release cytokines, promotion of angiogenesis and stimulation of extracellular matrix production [6].

Isoproterenol is a synthetic catecholamine and isoproterenol-induced myocardial injury is a standard experimental model for the investigation of pharmacological protective effects against ischemic injury [7]. The mechanism of isoproterenol-induced myocardial injury includes cytosolic calcium overload, lipid peroxide generation and procoagulant activity [8,9]. The pathological process of isoproterenol-induced myocardial damage is characterized by patchy areas of myocardial ischemia and this myocardial injury has many similarities to the traditional MI, attributed to ischemic coronary disease [10].

Interleukin-8, also known as CXCL-8, is a member of the CXC chemokine family which was originally discovered as a chemotactic factor for leukocytes [11]. IL-8 and the related cytokines are produced in several tissues upon inflammation, infection, ischemia and trauma [12]. Recent data has also implicated IL-8 as a mediator in human cancer progression through its potential function as a mitogenic, and angiogenic factor [11]. IL-8 was also found to directly promote angiogenesis by enhancing endothelial cell survival by inhibiting the apoptosis of endothelial cells resulting from inhibition of apoptotic response associated with increased levels of anti-apoptotic factors Bcl-xl and Bcl-2 [13].

There are no reports on the effect of exogenous IGF-1 on circulating IL-8 level and angiogenesis in the ischemic heart. We hypothesize that IGF-1 could play a protective role in myocardial ischemia by enhancing the circulating levels of angiogenic cytokine interleukin-8. Hence the present study was taken up with an objective to evaluate the effect of exogenous IGF-1 on circulating level of IL-8 and myocardial angiogenesis in experimentally-induced myocardial infarction in rats. We hypothesize that IGF-I protect ischemic myocardium with neovascularization by increasing circulating angiogenic factors.

## 2. Results and Discussion

### 2.1. Heart Weight

The Kruskal Wallis global comparison test revealed a statistically significant difference among all treatment groups. When compared in a pair-wise manner, the ISO-5d and ISO-10d group showed a significant increase in mean heart weight when compared to the control group. Heart weight was significantly higher (*p* < 0.05) in IGF-1 treatment group after 5th and 10th day respectively compared to control group. Treatment with IGF-1 to ISO groups significantly increased heart weight in both 5 days and 10 days treatment groups. Compared to 5 days treatment group, ISO-10d group had significantly reduced heart weight and in ISO with IGF-1 treated group, 10 days treatment had significant increase (*p* < 0.05) in heart weight (Table 1).

### 2.2. CK-MB

ISO-5d and ISO-10d groups showed a significant increase in mean CK-MB levels when compared to Control-5d and Control-10d groups respectively. A significant reduction (*p* < 0.05) in CK-MB levels was present in the ISO-10d group when compared to the ISO-5d group. There was no significant difference between the serum CK-MB levels between Control-5d, Control-10d, IGF-5d and IGF-10d groups. There was significant reduction in CK-MB levels in group ISO + IGF-5d when compared with group ISO-5d and ISO-10d groups. There was more significant reduction of CK-MB levels in group ISO + IGF-10d group when compared to the ISO-10d group (Table 2).

### 2.3. LDH

When compared in a pair-wise manner, groups ISO-5d and ISO-10d showed a significant increase (*p* < 0.05) in LDH levels when compared to Control-5d and Control-10d groups respectively. There was more significant increase in LDH level in ISO-10d group than ISO-5d. Statistical analysis showed no difference between LDH levels of groups control-5d and control-10d. The LDH levels in groups ISO + IGF-5d and ISO + IGF-10d were significantly lower (*p* < 0.05) when compared to ISO-5d and ISO-10d groups respectively. But the level remained significantly higher than control groups. Administration of IGF-1 decreased LDH levels significantly in MI rats but did not bring levels down to that of control groups (*p* < 0.05) (Table 2).

### 2.4. IGF-1

When compared in a pair-wise manner, groups ISO-5d and ISO-10d showed a significant increase (*p* < 0.05) in serum IGF-1 levels as compared to groups Control-5d and Control-10d respectively. There was more significant increase in IGF-1 in ISO-10d than ISO-5d group. Treatment with IGF-1 to ISO groups, further increased IGF-1 levels and ISO-10d with IGF-1 had significantly more IGF-1 level in serum than ISO-5d with IGF-1. IGF-1 alone group also had significantly higher (*p* < 0.05) IGF-1 level than control groups. IGF-1 treatment for 10 days significantly elevated serum IGF-1 levels than 5 days treatment (Table 3).

### 2.5. IL-8

The Kruskal Wallis test was used for global comparison and revealed a significant difference of in IL-8 levels among treatment groups. There was no significant change in IL-8 levels in control-5d and 10d groups. But ISO treatment for 5 and 10 days duration had significant effect on circulating IL-8 levels. There was a significant increase (*p* < 0.05) in IL-8 levels in both the groups with more significant increase in ISO-10d treatment. Concurrent treatment with IGF-1 for ISO groups further elevated serum IL-8 levels where 10 days IGF-1 treatment showed more significant increase than 5 days treatment (*p* < 0.05). Treatment with IGF-1 alone also significantly increased circulating IL-8 levels (Table 3).

### 2.6. Histopathological Changes

**Figure 1:**
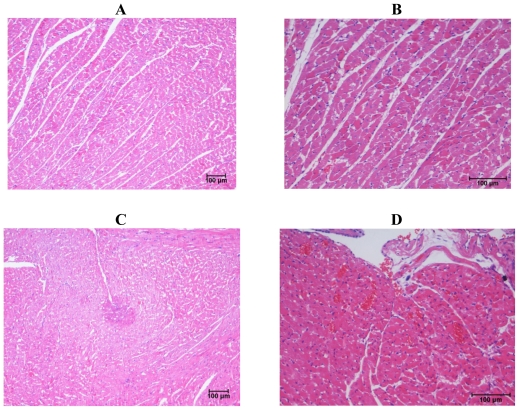
Photomicrographs of heart. (**A**) Normal heart from control group showing normal appearance of myocardial fibers in transverse section; (**B**) heart from IGF-10d group showing myocardium with adequate cellularity and normal morphology; (**C**) heart from ISO-5d showing a focally infarcted area surrounded by a zone of coagulative necrosis with numerous necrotic myocytes and scattered granulation tissue formation; (**D**) heart from ISO with IGF-1 for 10 days showing sprouting blood capillaries and extravasation of red blood cells between the myocardial muscle fibers.

The Control 5d and Control 10d groups showed histological evidence of robust myocardium architecture (Figure 1). The myocardium showed adequate cellularity and normal morphology. There were no features suggestive of myocyte necrosis, nuclear pyknosis, vascular proliferation, inflammatory cell infiltration, fibrosis or myocyte hypertrophy in this group. The 5d ISO group showed morphological changes in the myocardium suggestive of isoproterenol induced myocardial injury. There was strong evidence of myocyte necrosis and microscopic features were those of acute myocardial infarction showing neutrophilic infiltrate along with areas of necrosis, diffuse interstitial edema and pale myocytes with fading nuclei and decreased striations. The 10 day isoproterenol group showed morphological changes similar to that of the 5 day isoproterenol group (Table 4); however, there was significant increase in macrophage activity, red blood cell extravasation and scar formation. Histological analysis of samples from IGF-1 alone groups showed normal myocardium morphology. There was mild vascular proliferation evident as areas of tiny new blood vessels lined by thin endothelium. Histological analysis of the samples from ISO with IGF-1 groups revealed varying amounts of myocardium pathology. Evidence of myocyte necrosis and nuclear pyknosis were observed but to a lesser degree when compared to ISO alone treated samples. These changes were accompanied by the usual inflammatory cell infiltrate, edema and muscle hypertrophy (Table 4). Numerous areas of capillary sprouting were seen in the necrotic areas throughout the sample fields and more prominent neovascularization were observed when compared to other groups.

## 3. Discussion

Our research study showed that in rats, administration of isoproterenol (ISO; Sigma^®^; 85 mg/kg b. wt.) on two consecutive days has resulted in severe myocardial injury which was confirmed by elevations in CK-MB and LDH levels. This finding is in accordance with previous studies that has used isoproterenol-induced myocardial injury as an experimental model for the investigation of pharmacological protective effects of several drugs against ischemic injury [14,15]. We found severe cellular morphological alterations in our histopathological analysis in the myocardium as have been shown in previous studies [16]. These changes correspond to a significant elevation in serum cardiac marker levels confirming myocardial infarction. The ISO treatment produced functional alterations to the myocytes that manifested as an increase in serum cardiac biomarkers (CK-MB and LDH) levels. The biochemical results described in this study are in consonance with earlier reports on isoproterenol-induced myocardial injury; in the form of increased cardiac biomarker levels [9]. Injury to myocytes caused the release of cardiac enzymes and these enzymes leaked into the circulation elevating their serum concentrations. Panda and Naik, [16] in their research study reported that there was significant alteration in biochemical parameters (increased levels of AST, LDH and CK-MB in serum) with the induction of myocardial necrosis using ISO. In our study, more significant elevation of the cardiac enzymes were seen 24 h after the last dose of ISO, and both 5 and 10 days of treatment groups still showed increased CK-MB and LDH levels more than control animals, indicating a severe myocardial damage with ISO treatment.

We demonstrated that ISO treatment caused a significant increase in the heart weight when compared to the control group and similar finding were found in rats with MI that received IGF-1 treatment. This finding is consistent with findings of a study conducted by Boucher *et al*. [17] although they reported that IGF-1 drug administration normalized cardiac hypertrophy which we did not discover in our study. This could possibly be explained by the fact that significant ventricular hypertrophy developed through the excessive positive inotropic effect caused by ISO as previously described. IGF-1 alone treatment in normal rats did not cause a significant increase in heart weight although mild myocyte hypertrophy was observed from our histopathological analysis.

The complex metabolic and morphological changes in the myocardium caused by ISO treatment were possible via a variety of mechanisms that leads to necrosis. Zhang *et al*. [18] has shown that high-dose ISO produces relative hypoxia leading to ischemia by excessive activation of β1-adrenergic receptors leading to increased inotropic, chronotropic and dromotropic effects. Another mechanism is possibly by the formation of reactive oxygen species (ROS) through the auto-oxidation of ISO leading to peroxidative damage [19,20]. ISO is also thought to have procoagulant activity which predisposes to the thrombotic phenomena and aggravates ischemic myocardial damage as has been shown in a previous study by Pinelli *et al*. [9].

The present study showed that circulating levels of interleukin-8, an angiogenic cytokine and the proangiogenic growth factor IGF-1 were significantly increased after isoproterenol treatment. Ischemic injury is known to cause the release of angiogenic factors such as cytokines and vascular growth factors derived from the local tissue area and the blood circulation [5]. Our findings have shown that ischemic injury caused by ISO has caused increased secretion of both IGF-1 and IL-8. This could be an adaptive response of the myocardium for repair and revascularization to maintain oxygen supply after isoproterenol-induced myocardial hypoxia [21]. As have been reported by Folkman *et al*. [22] physiological response to the development of tissue ischemia includes the up-regulation of angiogenic growth factors and mobilization of circulating cellular elements that together enable development of an accessory vasculature.

Rabinovsky *et al*. [6] has also reported that angiogenesis occurs after ischemia stimulated by circulating angiogenic factors that helps in the regulation of endothelial cell migration, cell survival and endothelial cell proliferation. The findings of our histopathological analysis has clearly supported this view as there was a mild to moderate degree of vascular proliferation and neovascularization in the regions of the myocardium that has succumbed to ISO-induced myocardial necrosis, supporting our *in vivo* biochemical data on the concomitant rise in circulating angiogenic factors that may perhaps play a role in the stimulation of neovascularization.

From our present study, significant increase in circulating levels of IL-8 was observed after insulin like growth factor-1 treatment in normal rats as compared to control rats. Insulin like growth factor is a potent angiogenic factor [6,23,24] that promotes angiogenesis in several physiological and pathological conditions. Boucher *et al*. [17] has shown that IGF-1 has pro-angiogenic effects even at a low dose as low as 1 μg/kg. IGF-1 is postulated to induce angiogenesis through interaction with locally produced angiogenic factors [25]. IGF-1 may increase the release of other endothelial cell-derived angiogenic factors into the circulation when this factor binds to its receptors on endothelial cells [26]. Our findings confirm that one of the mechanisms by which IGF-1 promotes angiogenesis maybe via the stimulation of release of angiogenic, cytokine IL-8. IGF is known to have cardioprotective action during myocardial ischemia, but the mechanism of this protection is not clear yet [27]. One of the mechanism by which IGF protects myocardium during ischemia could be through stimulation of angiogenesis. Histopathological findings in the present study confirm the vasculogenesis effect of IGF-1. With increased IL-8 release there was synergistic effect of IGF-1 to increase the proliferation of endothelial cells, migration and survival to form new blood vessels [25].

IL-8 and the related cytokines are produced in several tissues upon inflammation, infection, ischemia and trauma [12]. The cytokine IL-8 was previously found to be a macrophage-derived proangiogenic factor causing proliferation and chemotaxis of endothelial cells [28]. Recently, IL-8 has been considered as mediator in cancer progression through its mitogenic, and angiogenic functions [11]. The present *in vivo* biochemical and histopathological data support these findings from previous studies that these angiogenic factor parameters possibly play a role in neovascularization in the ischemic myocardium by possibly stimulating endothelial cell proliferation and migration; which are thought to be the most important roles of these angiogenic factors. IL-8 was found to function as a macrophage-derived pro-angiogenic factor, a potent mediator of angiogenesis and this property was first demonstrated in the early 1990s when the recombinant form of IL-8 was shown to have chemotactic properties on endothelial cells [28]. Histological findings of neovascularization with IGF-treatment and biochemical increase in IL-8 in IGF-1 treated rats in the present study, confirmed the angiogenesis role of this cytokine.

In this study, we have also looked into the effect of difference in duration (5 and 10 days) of IGF-1 treatment on circulating IGF-1 and IL-8 level. Our *in vivo* biochemical data analysis revealed that IGF-1 stimulated a significant increase in IL-8 after 5 and 10 days treatment with IGF-1. The results showed that a longer duration (10 days) of IGF-1 treatment in rats with MI was more beneficial in increasing circulating the angiogenic cytokine IL-8 and bringing about vascular proliferation in myocardium as compared to the 5 day treatment group. The improved angiogenic factor parameter profile in ISO + IGF-1 10 day treatment group compared to the 5 day group could be explained by the fact that IGF-1 continued to stimulate angiogenic factor production and in synergy with the normal pathophysiological response to increase angiogenic factor production during ischemic injury in the myocardium for repair and revascularization.

## 4. Experimental Section

### 4.1. Chemicals

Isoproterenol hydrochloride (ISO), Insulin like growth factor-1 (IGF-1), were purchased from Sigma-Aldrich (St. Louis, MO). All other chemicals were obtained from local sources and were of analytical grade. IGF-1, IL-8 assay kits were purchased from USCN Life Science (USCN Life Science and Technology, China). CK-MB and lactate dehydrogenase assay kits were obtained from BioAssay Systems (USA).

### 4.2 Animals

Sixty four, adult male Sprague-Dawley rats (12 weeks old, 250–280mg body weight) were used for this study. The animals were kept at standard laboratory conditions on arrival with temperature 25 ± 2 ºC and 12 h/12 h light-dark cycle. Standard rodent feed and water were available ad libitum. The animals were kept for one week and allowed to acclimatize to the laboratory conditions before the commencement of the experiment.

The rats were randomly divided into eight groups with eight animals in each group.

Group 1—Control group: 5 days
Group 2—Control group: 10 days
Group 3—IGF-1 alone: 5 days
Group 4—IGF-1 alone: 10 days
Group 5—ISO alone: 5 days
Group 6—ISO alone: 10 days
Group 7—ISO with IGF-1: 5 days
Group 8—ISO with IGF-1: 10 days

Rats in group 1 and 2 were kept under standard laboratory conditions and treated with saline only, subcutaneously for 5 and 10 days. Rats in group 5 and 6 received isoproterenol only, subcutaneously at a dose of 85mg/kg body weight at an interval of 24 h for 2 days to induce myocardial ischemia. They were maintained under standard laboratory conditions without any further treatment for 5 and 10 days. Rats in group 7 and 8 were administered isoproterenol subcutaneously for two days (85 mg/kg BW) followed by treatment with IGF-1 at a dose of 2 μg/rat/day for 5 and 10 days. All the experimental procedures were conducted daily between 9:00 am–11:00 am and the experimental protocol was approved by Institutional research and ethics committee. At the end of experimental duration (on day 6 and 11), the body weight of the animals were recorded and the rats were sacrificed by overdose of anaesthesia. Blood samples were collected by cardiac puncture and heart was dissected out, washed in cold buffer and weighed. Blood was centrifuged and serum was separated and stored at −80 °C for biochemical assays. Heart was kept in formalin (10%) for histopathological analysis.

### 4.3 Biochemical Assay

#### 4.3.1. Cardiac Biomarker Assay

CK-MB test is based on the principle of a solid phase enzyme linked immunosorbent assay. This test is based on the principle of a solid phase enzyme-linked immunosorbent assay. The assay system utilizes a monoclonal antibody directed against a distinct antigenic determinant on the CK-MB molecule and this antibody is pre-coated in the wells on the plate (solid phase). The antibody-enzyme conjugate consists of goat anti-CK-MB antibody conjugated to horseradish peroxidase (HRP). The test sample is allowed to react simultaneously with the two antibodies, resulting in the CK-MB molecules being sandwiched between the solid phase and enzyme-linked antibodies. After the specified incubation the wells are washed to remove unbound labeled antibodies and TMB (3,3′5,5′-tetramethyl-benzidine) reagent is added resulting in the development of a blue color. The color development is stopped using a stop solution, changing the color to yellow. The concentration of CK-MB is directly proportional to the intensity of the color in the wells. Absorbance is measured spectrophotometrically using a 450 nm absorbance filter. CK-MB concentrations are expressed in ng/mL.

#### 4.3.2. LDH Assay

Lactate dehydrogenase is an oxidoreductase which catalyses the interconversion of lactate and pyruvate. This assay is based on the reduction of the tetrazolium salt MTT in a NADH-coupled enzymatic reaction to a reduced form of MTT which exhibits an absorption maximum at 565 nm. The intensity of the purple color formed is directly proportional to the enzyme activity. The plate was read at 0 min and again after 25 min on a plate reader. Upon spectrophotometric analysis, LDH concentration is calculated using the equation provided in the kit manual. LDH concentrations are expressed as IU/L.

#### 4.3.3. IGF-1 Assay

The USCN Life IGF-1 ELISA kit was used for the determination of blood IGF-1 levels from serum samples. The microtitre plate provided in this kit has been pre-coated with an antibody specific to IGF-1. Standards or samples are then added to the appropriate microtitre plate wells with a biotin-conjugated polyclonal antibody preparation specific for IGF-1 and Avidin conjugated to horseradish peroxidase (HRP) is added to each microtitre well and incubated. Then a TMB (3,3′5,5′-tetramethyl-benzidine) substrate solution is added to each well. Only those wells that contain IGF-1, biotin-conjugated antibody and enzyme conjugate Avidin will exhibit a change in color. The enzyme-substrate reaction is terminated by the addition of a sulfuric acid solution and the color change is measured spectrophotometrically at a wavelength of 450 nm. The concentration of IGF-1 in the samples was expressed as ng/mL.

#### 4.3.4. IL-8 Assay

The microtitre plate provided in this kit has been pre-coated with an antibody specific to IL-8. Standards or samples are then added to the appropriate microtitre plate wells with a biotin-conjugated polyclonal antibody preparation specific for IL-8 and Avidin conjugated to horseradish peroxidase (HRP) is added to each microtitre well and incubated. Then a TMB (3,3′5,5′-tetramethyl-benzidine) substrate solution is added to each well. Only those wells that contain IL-8, biotin-conjugated antibody and enzyme conjugate Avidin will exhibit a change in color. The enzyme-substrate reaction is terminated by the addition of a sulfuric acid solution and the color change is measured spectrophotometrically at a wavelength of 450 nm. The concentration of IL-8 is expressed as pg/mL.

### 4.4. Histopathological Analysis

The heart tissue was processed into paraffin blocks. Tissue sections of 5 μm were then cut from the paraffin blocks using a rotary microtome and adhered on to microscopic slides. The slides were dewaxed and stained with hematoxylin-eosin (H&E) stain before they were mounted with cover slips. A minimum of 5 fields from every slide were evaluated semi-quantitatively for morphological changes expected in myocardial infarction such as edema, tissue necrosis, fibrosis, and angiogenesis using the Nikon Microscope ECLIPSE 80i. A histopathologist blinded to the study was assigned to analyze and grade the histological changes of each group using an ordinal scoring method where (− = no change, + = mild change, ++ = moderate change and +++ = severe change).

### 4.5. Statistical Analyses

All analytical data were expressed in means ± SD. The level of significance was determined through the *p*-value and the *p*-value was set at 0.05. Global comparisons among all the groups were done using the Kruskal Wallis non-parametric test and pair-wise comparison between different groups was done using the Mann-Whitney-U test. Comparison was done to explore the effect of ISO and also the nullifying effect of IGF-1 on ISO-induced myocardial infarction.

## 5. Conclusions

Thus in conclusion, insulin like growth factor-1 significantly increased circulating angiogenic cytokine IL-8. There was significant cardioprotection provided by IGF-1 with stimulation of endothelial proliferation and survival as evidenced by neovascularization with 10 days of treatment. The deleterious effects of isoproterenol on the myocardium were significantly protected by IGF-1 treatment. Thus, IGF-1 and cytokine IL-8 may be considered as a novel and efficient therapeutic strategy for myocardial infarction in humans by enhancing angiogenesis, leading to chronic improvement in cardiac function.

## Figures and Tables

**Table 1:** Effect of Insulin like growth factor-1 (IGF-1) on heart weight (g).

Groups	Heart Weight (g)
Control: 5 days	0.263 ± 0.040
Control: 10 days	0.237 ± 0.081
IGF-1 alone: 5 days	0.352 ± 0.033 x
IGF-1 alone: 10 days	0.333 ± 0.092 a,♦
Isoproterenol: 5 days	0.349 ±0.051 x
Isoproterenol: 10 days	0.317 ±0.037 a,b,♦
ISO + IGF-1: 5 days	0.378 ± 0.023 x,y,z
ISO + IGF-1: 10 days	0.394 ± 0.052 a,b,c,♦

Values are means ± standard deviation;

a *p* < 0.05, significantly different from control 10 days;

b *p* < 0.05, significantly different from IGF-1 alone 10 days;

c *p* < 0.05, significantly different from ISO 10 days;

x *p* < 0.05, significantly different from control 5 days;

y *p* < 0.05, significantly different from IGF-1 alone 5 days;

z *p* < 0.05, significantly different from ISO 5 days;

♦ *p* < 0.05, 5 days *vs.* 10 days.

**Table 2:** Effect of IGF-1 on Cardiac Markers.

Groups	CK-MB (ng/mL)	LDH (IU/L)
Control: 5 days	2.456 ± 0.021	23.182 ± 1.872
Control: 10 days	3.003 ± 0.055 ♦	24.363 ± 1.830
IGF-1 alone: 5 days	3.632 ± 0.061 x	29.111 ± 1.770 x
IGF-1 alone: 10 days	3.776 ± 0.042 a	31.300 ± 2.991 a
Isoproterenol: 5 days	5.832 ± 0.059 x,y	65.767 ± 2.003 x,y
Isoproterenol: 10 days	5. 692 ± 0.092 a,b,♦	79.605 ± 2.600 a,b,♦
ISO + IGF-1: 5 days	3.823 ± 0.066 x,y,z	39.993 ± 1.446 x,y,z
ISO + IGF-1: 10 days	3.727 ± 0.052 a,c	42.550 ± 2.001 a,b,c

Values are means ± standard deviation;

a *p* < 0.05, significantly different from control 10 days;

b *p* < 0.05, significantly different from IGF-1 alone 10 days;

c *p* < 0.05, significantly different from ISO 10 days;

x *p* < 0.05, significantly different from control 5 days;

y *p* < 0.05, significantly different from IGF-1 alone 5 days;

z *p* < 0.05, significantly different from ISO 5 days;

♦ *p* < 0.05, 5 days *vs*. 10 days.

**Table 3:** Effect of IGF-1 on circulating IL-8 level.

Groups	IGF-1 (ng/mL)	IL-8 (pg/mL)
Control: 5 days	3.666 ± 0.234	65.004 ± 0.894
Control: 10 days	3.529 ± 0.564	70.523 ± 0.777
IGF-1 alone: 5 days	6.873 ± 0.077 x	78.865 ± 0.538 x
IGF-1 alone: 10 days	7.666 ± 0.058 a,♦	82.566 ± 0.662 a,♦
Isoproterenol: 5 days	5.143 ± 0.196 x,y	89.228 ± 0.009 x,y
Isoproterenol: 10 days	6.624 ± 0.404 a,b,♦	93.046 ± 0.504 a,b,♦
ISO + IGF-1: 5 days	7.836 ± 0.950 x,z	103.613 ± 0.442 x,y,z
ISO + IGF-1: 10 days	8.802 ± 0.773 a,b,c,♦	106.353 ± 0.770 a,b,c,♦

Values are means ± standard deviation;

a *p* < 0.05, significantly different from control 10 days;

b *p* < 0.05, significantly different from IGF-1 alone 10 days;

c *p* < 0.05, significantly different from ISO 10 days;

x *p* < 0.05, significantly different from control 5 days;

y *p* < 0.05, significantly different from IGF-1 alone 5 days;

z *p* < 0.05, significantly different from ISO 5 days;

♦ *p* < 0.05, 5 days *vs*. 10 days.

**Table 4:** Summary of semi-quantitative histological findings.

Groups	Myocyte Necrosis	Nuclear Pyknosis	Vascular Proliferation	Macrophage Activity	Scar Formation	Muscle Hypertrophy
Control: 5 days	−	−	−	−	−	−
Control: 10 days	−	−	−	−	−	−
IGF-1 alone: 5 days	−	−	+	−	−	+
IGF-1 alone: 10 days	−	−	+	−	−	+
Isoproterenol: 5 days	+++	++	−	+	++	++
Isoproterenol: 10 days	+++	++	+	++	+++	++
ISO + IGF-1: 5 days	++	+	++	++	+	++
ISO + IGF-1: 10 days	++	+	+++	+++	++	++

−: Designates *no* histopathological change; +: Designates *mild* histopathological change; ++: Designates *moderate* histopathological change; +++: Designates *severe* histopathological change.
